# Diagnostic glide including more drug categories and more drug induced morbidities in ICD-11 may muddle our understanding of addictive disorders

**DOI:** 10.1192/j.eurpsy.2025.75

**Published:** 2025-08-26

**Authors:** J. G. Bramness

**Affiliations:** UiT The Arctic University of Norway, Tromsø, Norway

## Abstract

**Abstract:**

In the ICD-11 the number of drug categories as “headers” for the substance related morbidity chapters has doubled. Furthermore, the ICD-11 has rationalized the number of criteria for substance dependence from six to three, but in the process in fact the threshold for receiving a diagnosis of substance dependence has been reduced substantially. Lastly, the ICD-11 has increased substantially the number of drug induced comorbidities. The former “substance induced psychosis” has now been joined by substance induced depression, anxiety disorder and even OCD for some drugs. This talk will raise some questions regarding the functionality of these changes.

**Disclosure of Interest:**

None Declared

